# Editorial: Thinking Through the Schizophrenia Spectrum: Nosological Scenarios and Perspectives Beyond Psychosis

**DOI:** 10.3389/fpsyt.2021.772307

**Published:** 2021-10-05

**Authors:** Anna Comparelli, Mads Gram Henriksen, Andrea Raballo

**Affiliations:** ^1^Department of Psychiatry, Sant'Andrea Hospital of Rome, Rome, Italy; ^2^Department of Communication, Center for Subjectivity Research, University of Copenhagen and Mental Health Center Amager, Copenhagen, Denmark; ^3^Section of Psychiatry, Clinical Psychology and Rehabilitation, Department of Medicine, University of Perugia, Perugia, Italy; ^4^Center for Translational, Phenomenological and Developmental Psychopathology, Perugia University Hospital, Perugia, Italy

**Keywords:** schizophrenia, psychosis spectrum, nosology, endophenotype, neurodevelopment

The concept of schizophrenia remains a matter of enduring debate, although often limited to the psycho-behavioral surface of its descriptive criteria. This Research Topic integrates such debate providing a set of different state of the art perspectives on the challenges surrounding the schizophrenia concept.

In contemporary research, the notion of a broad psychotic spectrum, within which schizophrenia loses its nosological boundaries and dissolves, has gained once again a certain momentum. Proponents of this view argue that this concept is better supported by genetic, neurobiological, and neurodevelopmental data than the traditional categorically defined diagnoses. Others, however, defend the concept of schizophrenia or schizophrenia spectrum and continue the search for its essence that will allow demarcation of schizophrenia from other forms of psychosis. From yet other points of view, the Research Domain of Criteria (RDoC) and the Hierarchical Taxonomy of Psychopathology (HiTOP) address the general validity crisis of current nosography and explore new possible nosological horizons. This Research Topic aimed at an overview of possible nosological scenarios for schizophrenia and its spectrum disorders.

Consistent with the RDoC approach, Cuthbert and Morris, in their perspective article, argue that genomic data provide increasing support for the concept of systematic, trans-diagnostic components of neurodevelopmental and genomic spectra. In this view, the neurodevelopmental gradient is not simply a matter of cognitive performance, but a result of multiple functional domains, whose combinations comprise potentially significant clinical phenotypes with schizophrenia representing one segment of multiple broader spectra.

From the perspective of HiTOP, Cowan and Mittal, in their brief research report article, present a transdiagnostic dimensional analysis of psychiatric comorbidity in a Clinical High Risk (CHR) sample. They found that, although the CHR group presented more positive symptoms compared to healthy controls, the negative symptom factor was much more strongly linked than the other factors to impaired cognition, impaired social and role functioning, and risk of transition to psychosis This finding suggests that negative symptoms may be more specific of the progression toward psychosis in the broader spectrum of subthreshold positive psychopathology.

In their original research article, Pontillo et al. found that in children and adolescents with early and very early onset schizophrenia, the presence or absence of neurodevelopmental disturbances or difficulties differentiated the characteristics of psychotic onset. In fact, in the presence of neurodevelopmental dysfunctions, the onset occurs earlier and was associated with more severe functional impairment, positive and disorganized symptoms. By contrast, in children and adolescents without neurodevelopmental disorders or difficulties, the psychotic onset was later and associated with negative symptoms.

Collectively, these papers explore the relationship between neurodevelopmental disorders and schizophrenia through analysis of psychopathological trajectories and clinical pathways of childhood neuropsychiatric disorders.

Exploring new potential phenotypes and endophenotypes, was another aim of the Research Topic, and it was addressed by Gao et al. In their original research article, patients with schizophrenia showed an association between cognitive dysfunction and increased regional homogeneity values (ReHo), an index of neural activity, in prefrontal regions including the right rectus gyrus, inferior frontal gyrus/insula, the lower right and left insula, interestingly, all in the limbic area. Furthermore, ReHo values in the right inferior frontal gyrus/insula were correlated with negative symptoms and verbal learning tasks. The combined increases of ReHo values in the left inferior frontal gyrus/insula with the right gyrus rectus may be an underlying biomarker differentiating patients with schizophrenia from healthy controls.

Bleuler, who coined the schizophrenia concept, considered formal thought disorders (“disturbances of association”), and ego-disorders as fundamental symptoms of schizophrenia. In contemporary psychopathological research, ego-disorders have been re-conceptualized and systematically assessed under the notion of self-disorders. The novelty of the original research paper by Nordgaard et al. is the finding of a close relationship between formal thought disorders and self-disorders—a finding that further reinforces the notion of self-disorders as a unifying, psychopathological core beneath the apparently heterogeneous symptoms of schizophrenia spectrum disorders.

Consistently, through the novel theoretical approach of shared intentionality, Salice and Henriksen, in their Hypothesis and Theory Article, aimed to differentiate the sources of social difficulties in schizophrenia spectrum disorder and severe autism spectrum disorder. They proposed a distinction between two kinds of shared intentionality—joint- and we-intentionality—and argue that we-intentionality may be affected in schizophrenia, whereas both joint- and we-intentionality are impaired in autism. They argue that the qualitatively distinct social difficulties are linked to the disorders' different psychopathological cores. Trait-like self-disorders may affect the psychological preconditions for we-intentionality, whereas the psychological preconditions for both forms of shared intentionality are impeded by problems with the ability to “be moved” by others' intentions, perspective-taking, and mind-reading in autism.

The complex relationship between formal thought disorders, self-disorders, negative symptoms, and social cognition may be reflected in a specific phenotype, which, in part, concerns dis-sociality and detachment from the common sensical world. This phenotype is reflected in the classical phenomenon of schizophrenic autism, which Bleuler also originally described as a fundamental symptom of schizophrenia. In their original research article, Palumbo et al. proposed a novel scale, viz. the Autism Rating Scale (ARS), to detect and measure the phenomenon of schizophrenic autism. Their article explored the psychometric properties of the ARS and furthermore found that scorings on the ARS differentiated patients with schizophrenia and bipolar disorder.

Finally, in their opinion article, Guloksuz and van Os provocatively restate their belief in the death of schizophrenia concept and in the promise of the wider psychosis spectrum concept. They summarize shortcomings to the schizophrenia concept (e.g., lack of etiological and phenotypic specificity as well as its stigmatizing connotations such as chronicity and deterioration) and highlight the benefits of the psychosis spectrum concept in conjunction with clinical characterization.

Overall, we hope that this Research Topic dedicated to the nosological promise and perils of the Schizophrenia Spectrum concept will contribute to further advancements in the field.

## Author Contributions

AC, MH, and AR contributed equally to drafting the Editorial and revising it critically. All authors contributed to the article and approved the submitted version.

## Conflict of Interest

The authors declare that the research was conducted in the absence of any commercial or financial relationships that could be construed as a potential conflict of interest.

## Publisher's Note

All claims expressed in this article are solely those of the authors and do not necessarily represent those of their affiliated organizations, or those of the publisher, the editors and the reviewers. Any product that may be evaluated in this article, or claim that may be made by its manufacturer, is not guaranteed or endorsed by the publisher.

